# Resveratrol alleviates DSS-induced IBD in mice by regulating the intestinal microbiota-macrophage-arginine metabolism axis

**DOI:** 10.1186/s40001-023-01257-6

**Published:** 2023-09-02

**Authors:** Xinwei Xu, Dickson Kofi Wiredu Ocansey, Bing Pei, Yaqin Zhang, Naijian Wang, Zengxu Wang, Fei Mao

**Affiliations:** 1 Key Laboratory of Medical Science and Laboratory Medicine of Jiangsu Province, School of Medicine, Jiangsu University, Zhenjiang, 212013 Jiangsu People’s Republic of China; 2 School of Materials Science and Engineering, Jiangsu University, Zhenjiang, 212013 Jiangsu People’s Republic of China; 3 The Affiliated Suqian First People’s Hospital of Nanjing Medical University, Suqian, 223800 Jiangsu People’s Republic of China; 4 Zhenjiang Hospital of Chinese Traditional And Western Medicine, 18 Tuanshan Road, Runzhou District, Zhenjiang, 212000 Jiangsu People’s Republic of China

**Keywords:** Inflammatory bowel disease, Resveratrol, Microbiota, Arginine metabolism, Macrophage

## Abstract

**Background:**

Inflammatory bowel disease (IBD) is a global disease with a growing public health concern and is associated with a complex interplay of factors, including the microbiota and immune system. Resveratrol, a natural anti-inflammatory and antioxidant agent, is known to relieve IBD but the mechanism involved is largely unexplored.

**Methods:**

This study examines the modulatory effect of resveratrol on intestinal immunity, microbiota, metabolites, and related functions and pathways in the BALB/c mice model of IBD. Mouse RAW264.7 macrophage cell line was used to further explore the involvement of the macrophage-arginine metabolism axis. The treatment outcome was assessed through qRT-PCR, western blot, immunofluorescence, immunohistochemistry, and fecal 16S rDNA sequencing and UHPLC/Q-TOF–MS.

**Results:**

Results showed that resveratrol treatment significantly reduced disease activity index (DAI), retained mice weight, repaired colon and spleen tissues, upregulated IL-10 and the tight junction proteins Occludin and Claudin 1, and decreased pro-inflammatory cytokines IL-1β, IL-6, and TNF-α. Resveratrol reduced the number of dysregulated metabolites and improved the gut microbial community structure and diversity, including reversing changes in the phyla *Bacteroidetes*, *Proteobacteria*, and *Firmicutes*, increasing ‘beneficial’ genera, and decreasing potential pathogens such as *Lachnoclostridium*, *Acinobacter*, and *Serratia*. Arginine–proline metabolism was significantly different between the colitis-treated and untreated groups. In the colon mucosa and RAW264.7 macrophage, resveratrol regulated arginine metabolism towards colon protection by increasing Arg1 and Slc6a8 and decreasing iNOS.

**Conclusion:**

This uncovers a previously unknown mechanism of resveratrol treatment in IBD and provides the microbiota-macrophage-arginine metabolism axis as a potential therapeutic target for intestinal inflammation.

**Supplementary Information:**

The online version contains supplementary material available at 10.1186/s40001-023-01257-6.

## Background

Inflammatory bowel disease (IBD), which includes ulcerative colitis (UC) and Crohn’s disease (CD), is a global disease with growing public health concerns due to increasing incidence. In 2020, it was asserted that developing countries are in the emergence stage of IBD evolution, newly industrialized countries are in the Acceleration in Incidence stage, while Western countries are in the Compounding Prevalence stage [1]. Although the exact cause of IBD is unknown, it is believed to be associated with a complex interplay of microbiota, immune system, environmental, and genetic factors [2, 3]. IBD is linked with compositional and metabolic alterations in the intestinal microbiota (dysbiosis) coupled with immune dysregulation, where chronic inflammation shapes the gut microbiota and vice versa, contributing to the development and progression of IBD [4]. Therefore, the restoration of microbial composition and diversity, and their derived metabolites in the gut is a promising therapeutic approach in IBD.

Macrophages are the gatekeepers of intestinal immune homeostasis as they discriminate between innocuous antigens and potential pathogens to maintain tolerogenic immunity, thus, their impairment leads to chronic relapsing immune dysregulation and pathologies of the gastrointestinal tract, including IBD [5]. Large numbers of macrophages are found in the colon mucosa of IBD patients and animal models, participating in the initiation and resolution of inflammation. Macrophages are polarized into either pro-inflammatory M1 phenotypes via induction by interferon γ (IFN-γ), tumor necrosis factor-alpha (TNF-α), and bacterial lipopolysaccharide (LPS) to produce a wide range of proinflammatory cytokines such as interleukin (IL)-1β and IL-6, or anti-inflammatory M2 phonotypes via induction by IL-4 and IL-13 to express arginase 1 (Arg1) and anti-inflammatory cytokines, including IL-10 [6, 7]. In effect, M2 macrophages promote intestinal tissue repair and inflammation resolution and are found to also interact with the microbiota [8, 9]. The anti-inflammatory effect of macrophages is linked with arginine metabolism, as the two arginine catalytic enzymes, inducible nitric oxide synthase (iNOS) and Arg1, are well-characterized hallmark molecules of the classically and alternatively activated macrophages, respectively [10, 11]. In a related process, the depletion of intracellular creatine by downregulating the creatine transporter Slc6a8 (solute carrier family 6 member 8) alters macrophage-mediated immune responses since creatine inhibits iNOS by suppressing IFN-γ-JAK-STAT1 signaling and promotes IL-4-STAT6-activated Arg1 expression [11]. Because macrophages also express Arg1 and Slc6a8, a therapeutic substance that modulates arginine metabolism towards increases in Arg1 and Slc6a8, would be promising in the treatment of IBD.

As a potential therapy for IBD, resveratrol, a type of natural phenol and phytoalexin that acts against pathogens and possesses anti-inflammatory and antioxidant activity, has been widely studied [12, 13]. Resveratrol has been reported to relieve IBD in animal models by regulating immune responses and signaling pathways [14, 15] as well as the gut microbiome [16]. However, the mechanisms involved in these effects remain largely unknown. This study examines the treatment effect of resveratrol in the modulation of intestinal immune response, mucosal tissue repair, gut microbiota community structure and related functions, and metabolites and their associated pathways in the mice model of IBD. With the progress in next-generation sequencing technology, 16S rDNA of microbiota and UHPLC/Q-TOF–MS (ultra-high-performance liquid chromatography-quadrupole time-of-flight mass spectrometry) detection of metabolomic was used to identify various changes of the gut microbiota composition. The involvement of the macrophage-arginine metabolism axis was also examined.

## Material and methods

### Resveratrol

Resveratrol, a non-flavonoid polyphenol organic compound, is an antitoxin produced by many plants when stimulated. Its chemical name is 3,4',5-trihydroxy-1,2-diphenyl ethylene, and the chemical formula is C14H12O3. The resveratrol used in this study was purchased from Sigma, USA (CAS 501-36-0, PubChem Chemical No.24278055).

### Animal model

Male BALB/c mice (6–8 weeks old, 20 ± 2 g) were purchased from the Animal Research Center of Jiangsu University (Zhenjiang, China). All mice were treated accordingly in the SPF laboratory. Mice were randomly divided into 3 groups (*n* = 5/group); and the negative control group (NC group), the Dextran sulfate sodium (DSS)-induced colitis group (DSS group), and the Resveratrol-treated colitis group (RSV group). The NC group was given autoclaved purified water, while the DSS and RSV groups were given autoclaved purified water containing 3% DSS. In addition, mice in the RSV group were given resveratrol solvent (100 mg/kg) by oral gavage every day. The mice were weighed daily and their fecal characteristics were recorded. When the mice in the DSS group showed obvious bloody stool and weight loss, the feces of the mice in each group were collected aseptically for subsequent 16S rDNA sequencing and UHPLC/Q-TOF–MS. On the 10th day, all mice were sacrificed and colon and spleen tissues were collected for subsequent experiments.

### Cell culture

Mouse leukemic monocyte/macrophage cell line (RAW264.7) was purchased from Beiner Biotechnology Company (Beijing, China) and cultured in DMEM medium (Hyclone) containing 10% fetal calf serum (FBS; Excell,Uruguay) at 37 °C in humid air with 5% CO2. The RAW264.7 was cultured in an LPS-induced inflammatory environment and treated with or without resveratrol (80 nmol/ml) for 12 h.

### Western-blot

RIPA (Radio-Immunoprecipitation Assay) lysis buffer was added to colon tissue and RAW264.7 cells and the protein concentration was measured by the BCA (Bicinchoninic acid) method. A total of 200 μg of protein sample was separated by 10% SDS-PAGE. The isolated proteins were transferred onto PVDF membranes, blocked in 5% skim milk powder (dissolved in TBST buffer) for 1 h. PVDF membranes were incubated with primary antibodies (PCNA-1:1000, Abcam;Occludin-1:5000, Proteintech; Claudin1-1:1000, Proteintech; iNOS-1:500, Proteintech; Arg1-1:1000, Proteintech; Slc6a8-1:400, Proteintech) at 4 °C overnight, and then incubated with secondary antibodies at room temperature for 1 h to visualize protein bands and generate images.

### Quantitative Real-time (qRT)-PCR

Total RNA was extracted from mouse colons and RAW264.7 cells by chloroform extraction using Trizol reagent (Vazyme, Nanjing, China). cDNA was synthesized using the HiScript 1st Strand cDNA Synthesis Kit (Vazyme, Nanjing, China). β-actin was used as an internal control to detect the expression of target genes. Primer sequences used are shown in Table 1.

**Table 1:** Sequence of primers used for qRT-PCR

Primer name	Sequence (5' to 3')
mouse-IL-1β-F	CACTACAGGCTCCGAGATGAACAAC
mouse-IL-1β-R	TGTCGTTGCTTGGTTCTCCTTGTAC
mouse-IL6-F	CTCCCAACAGACCTGTCTATAC
mouse-IL6-R	CCATTGCACAACTCTTTTCTCA
mouse-TNF-α-F	ATGTCTCAGCCTCTTCTCATTC
mouse-TNF-α-R	GCTTGTCACTCGAATTTTGAGA
mouse-IL10-F	TTCTTTCAAACAAAGGACCAGC
mouse-IL10-R	GCAACCCAAGTAACCCTTAAAG
mouse iNOS-F	ATCTTGGAGCGAGTTGTGGATTGTC
mouse iNOS-R	TAGGTGAGGGCTTGGCTGAGTG
mouse Arg1-F	AATCTGGTTGTGTATCCTCGTT
mouse Arg1-R	AGAGGTGTATTAATGTCCGCAT
mouse Slc6a8-F	GCCCTACCTCCTCTCCTTCTTTCC
mouse Slc6a8-R	TTTCCCTCCTCTCCTGTTACCCAAG

### Immunohistochemistry (IHC)

Paraffin-embedded colon tissue Sections (4 μm thick) were processed for hematoxylin and eosin (H&E) staining or deparaffinized for immunohistochemistry (IHC). In IHC, sections were dewaxed and placed in 3% hydrogen peroxide solution, followed by incubation at room temperature for 30 min before thermal repair of antigens through boiling for 30 min in citrate buffer. After blocking with 5% BSA for 30 min, the primary antibody (Slc6a8- 1:200, Proteintech) was incubated at 4 °C overnight, followed by the secondary antibody (Wuhan Boster Biological Technology, Wuhan, China) at room temperature for 1 h. StreptAvidin Biotin Complex (SABC) was added and incubated at 37 °C for 30 min. Finally, diaminobenzidine substrate (DAB) was applied to sections and counter-stained with hematoxylin for microscopic examination.

### Immunofluorescence

The sections were dewaxed to block nonspecific antigens as described in IHC. Sections were incubated with iNOS antibody (1:200) and Arg1 antibody (1:300) at 4° overnight, and then incubated with the corresponding fluorescent secondary antibody for 2 h at room temperature. The specimens were stained with hoechest33342 (1:300; Sigma–Aldrich) for 10 min at room temperature and observed under a microscope. During the experiment, exposure to light was avoided to prevent fluorescence quenching.

### 16S rDNA gene sequencing

The experimental process for the fecal analysis of the microbiota community and function prediction involved extraction of genome DNA, amplicon generation, PCR products quantification and qualification, product mixing and purification, and library preparation and sequencing. The sample quality was strictly controlled in each link from DNA extraction to computer sequencing to ensure the authenticity of sequencing data. For the combined analysis of 16S metabolomics, the process involved 16S rDNA amplicon sequencing of significantly different microbiota and significantly different metabolites, followed by various correlation analyses. To confirm differences in the abundance of individual taxonomy between the groups, STAMP software was utilized. LEfSe was used for the quantitative analysis of biomarkers within different groups. To identify differences in microbial communities between the groups, ANOSIM and ADONIS were performed based on the Bray–Curtis dissimilarity distance matrices.

### LC–MS/MS Analysis

Analyses were performed using a UHPLC (1290 Infinity LC, Agilent Technologies) coupled to a quadrupole time-of-flight (AB SciexTripleTOF 6600) in Shanghai Applied Protein Technology Co., Ltd.

### Statistics Analysis

All data were expressed as mean ± SD. Comparisons between multiple groups were assessed by student *t*-test. *P* < 0.05 was considered statistically significant.

## Results

### Resveratrol relieves the features of colitis in mice

**Fig. 1:**
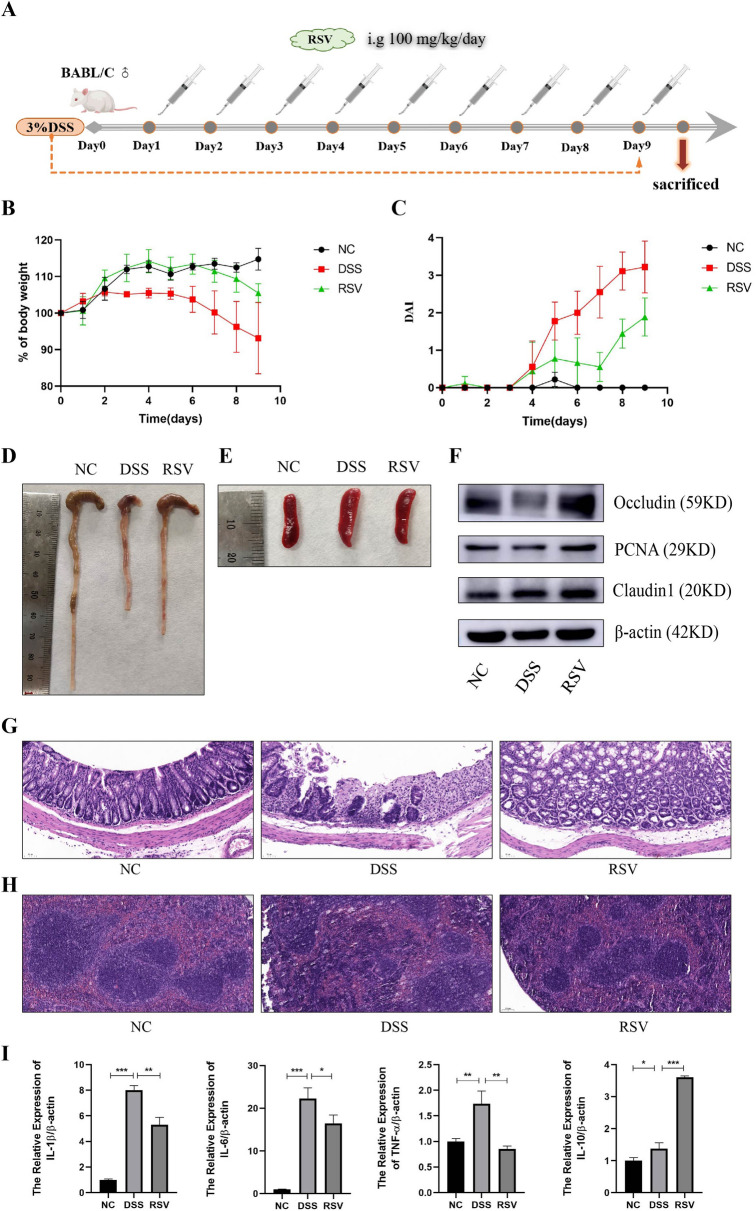
Resveratrol relieves the features of colitis in mice. **A** Schematic diagram of animal model construction. **B** Percentage of weight loss in each group during modeling. **C** Disease Activity Index score. **D:** General view of the colorectum. **E** General view of spleen. **F** Western blot detection of PCNA、Occludin、Claudin1 expression in colon tissues. **G** HE staining of colon tissue (200 ×). **H** HE staining of spleen tissue (100 ×). **I** qRT-PCR detection of cytokines expression in colon tissues. * represents *p* < 0.05, ** represents *p* < 0.01 and *** represents *p* < 0.001

The male BALB/c mice were randomly divided into 3 groups (*n* = 5/group); the negative control (NC) group, the DSS-induced colitis (DSS) group, and the Resveratrol-treated colitis (RSV) group. After the induction of colitis in the DSS and RSV groups, the RSV group was treated with 100 mg/kg resveratrol solvent by gavage every day (Fig. 1A). Weight assessment indicated that while the DSS group decreased in average weight, the RSV group significantly retained weight (Fig. 1B). The disease activity index (DAI) of the RSV group was reduced compared to the DSS group (Fig. 1C). Moreover, resveratrol treatment significantly restored the gross morphological appearance of mice colon and spleen tissues (Fig. 1D, E), as further confirmed with H&E staining (Fig. 1G, H) indicating reduced damage to intestinal villus structure in the RSV group relative to the DSS group. To examine whether these tissue and cellular changes translated into molecular level changes, we measured the expression level of the tight junction proteins Occludin and Claudin 1, as well as proliferating cell nuclear antigen (PCNA), an essential molecule in nucleic acid metabolism as a component of the replication and repair machinery. Results showed that resveratrol treatment increased the protein expression of Occludin, Claudin 1, and PCNA in mice colon compared to the untreated group (Fig. 1F). Cytokine analysis via qRT-PCR also showed increased anti-inflammatory cytokine IL-10 but decreased pro-inflammatory cytokines IL-1β, IL-6, and TNFα in the RSV group compared to the DSS group (Fig. 1I). These observations indicate the ability of resveratrol to reduce intestinal mucosal inflammation, repair tissue damage, and restore tight junction molecules, thus relieving the microscopic and macroscopic features of colitis in mice.

### Resveratrol improves the general community structure and diversity of gut microbiota

**Fig. 2:**
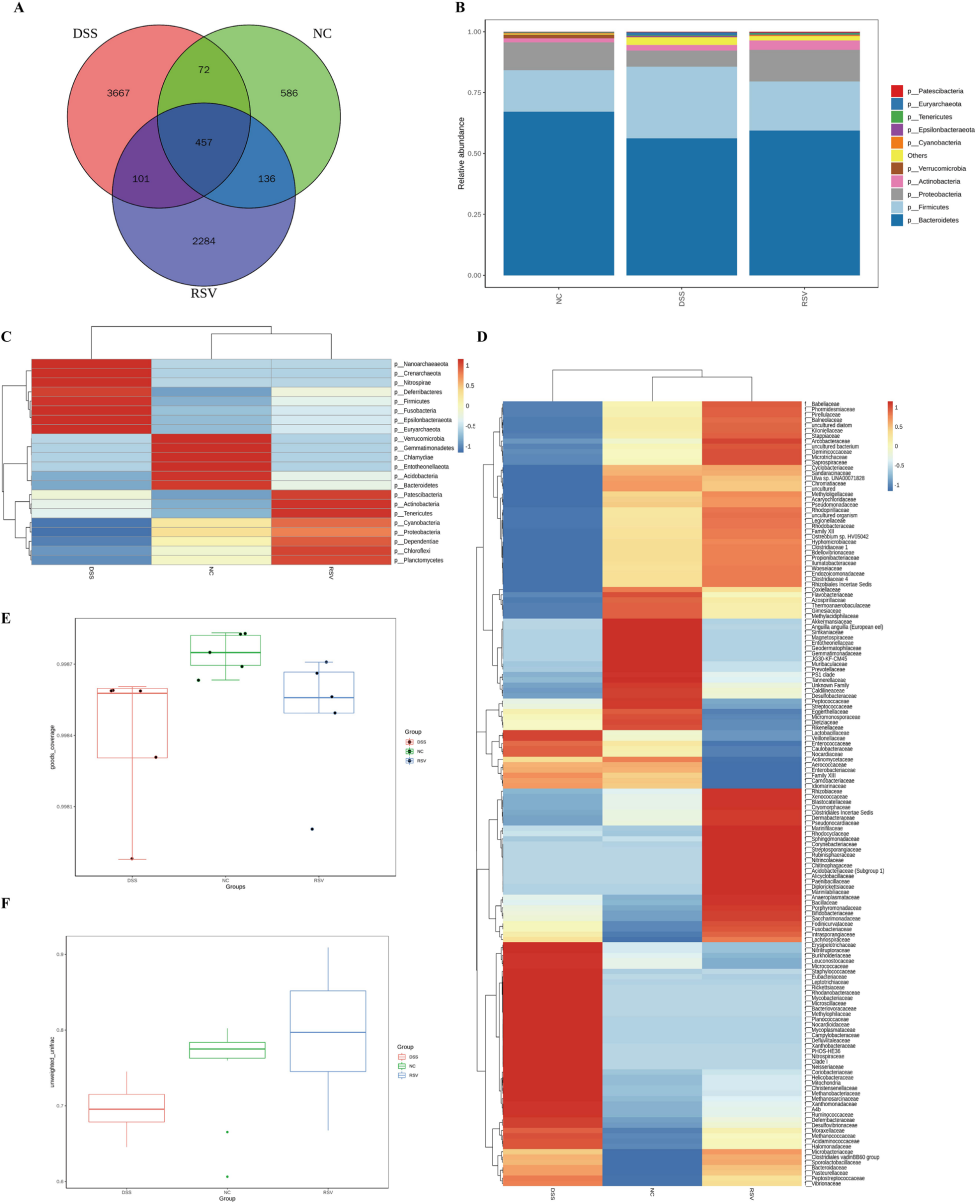
Resveratrol improves gut microbial general community structure and diversity. **A** Venn diagram of OTUs between the DSS group, the RSV group, and the control. **B** Community abundance of the top 10 phyla within the groups. **C** Cluster heatmap of species abundance at the family level within the groups. **D** Cluster heatmap of species abundance at the phyla level within the groups. **E** Goods coverage box chart of the difference between α diversity index of the groups. **F** Group difference analysis of β diversity based on Unweighted Unifrac distance

The effect of the treatment on the microbiota structure and diversity was examined through 16S rDNA gene sequencing of mice fecal samples. Quality control checks revealed adequacy of sample size and species richness, adequate sequencing data to sufficiently reflect the microbial information in the samples, and uniform distribution of species (Additional file 1: Fig. S1F–H). Venn analysis of OTUs showed that compared to the NC group (586 OTUs), the DSS group had increased OTU abundance (3667 OTUs), which was reduced in the RSV group (2284 OTUs). Moreover, the RSV group shared more common OTUs with the control group compared to the DSS group (136 vs 72) (Fig. 2A). Previous studies indicate that the phyla Firmicutes, Bacteroidetes, Proteobacteria, and Actinobacteria form approximately 99% of the microbiota, with Bacteroidetes and Firmicutes contributing about 90% [17, 18], thus, the *Bacteroidetes*/*Firmicutes* ratio is crucial in maintaining normal intestinal homeostasis. Our results showed that while the DSS group had reduced the abundance of *Bacteroidetes* and *Proteobacteria* and increased *Firmicutes*, resveratrol reversed these changes (Fig. 2B). The abundance of other phyla such as *Nanoarchaeaeota, Crenarchaeota, Nitrospirae, Deferribacteres, Fusobacteria, Epsilonbacteraeota*, and *Euryarchaeota* was also significantly restored by resveratrol (Fig. 2C). The cluster heatmap of abundant families further reveals the restoration effect of resveratrol treatment on the microbiota community structure as many clusters in the RSV group closely resemble the NC group compared to the DSS group (Fig. 2D). There was reduced alpha-diversity (Fig. 2E) in the DSS group (DSS vs NC *p*-value = 0.008) but a non-significant difference between the RSV and NC groups (p-value = 0.056). In addition, while there was a significant beta-diversity difference (Fig. 2F) between the DSS and NC groups (*p*-value = 0.026), there was a non-significant difference between the RSV and NC groups (*p*-value = 0.202). These findings show the ability of resveratrol to improve the colitis-induced microbiota community changes and significantly restore the alpha and beta diversity.

### Resveratrol modulates specific bacteria species and restores functional dysregulation

**Fig. 3:**
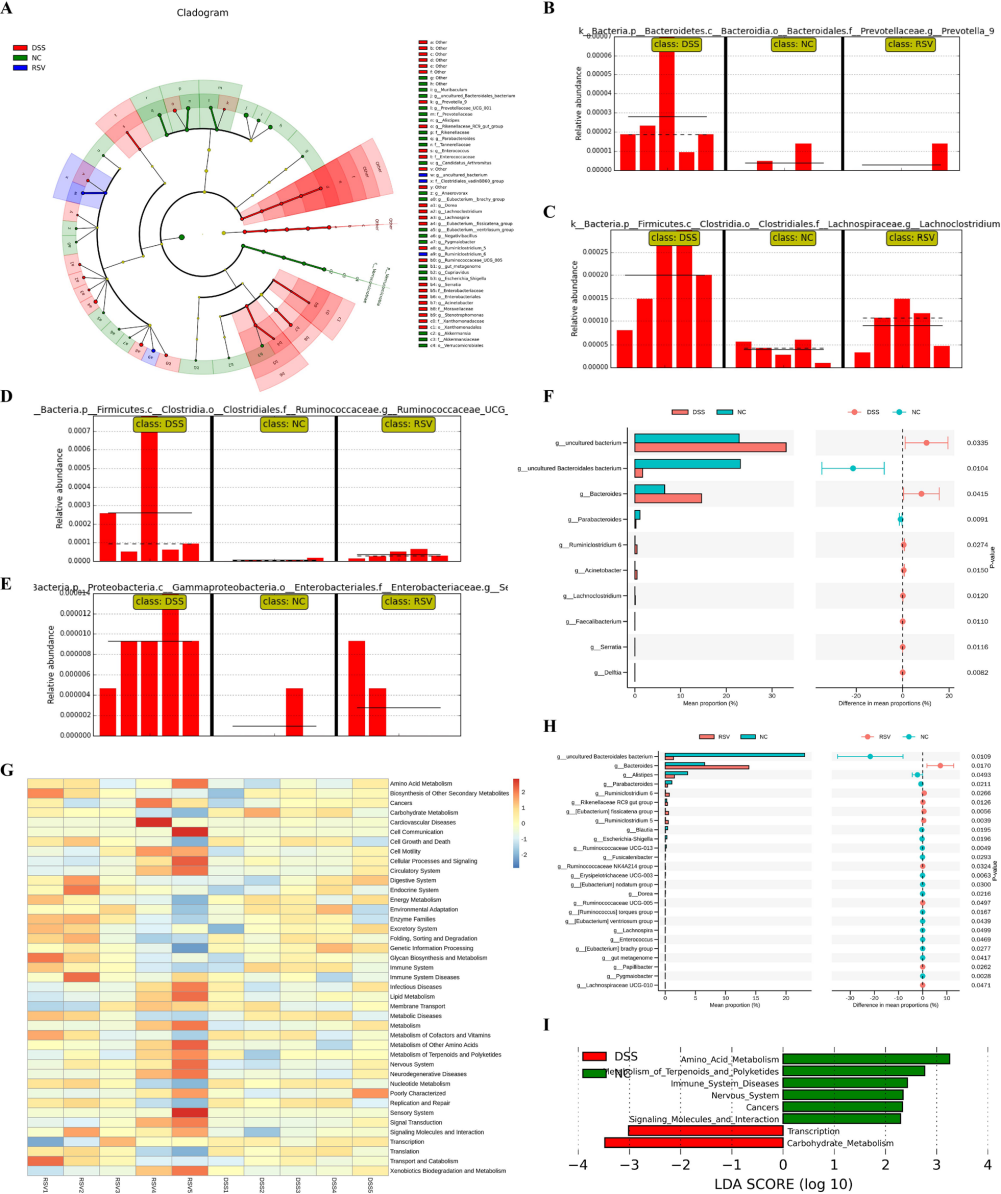
Resveratrol modulates specific bacteria species and restores functional dysregulation. **A** Cladogram (evolutionary branch diagram) of statistically different microbiota within each group. **B**–**E** Comparison of abundances of bacterial markers with significant differences between NC, DSS, and RSV groups (*Provotella_9,Lachnoclostridium, Ruminococcaceae_UCG_005, and Serrati*a). **F** STAMP t-test of species with significant differences at the genus level between the DSS and NC groups. **G** KEGG heatmap prediction of the function of differential bacteria between the RSV and DSS group. **H** STAMP t-test of species with significant differences at the genus level between the RSV and NC groups. **I** KEGG LDA score of predicted functions of the DSS vs NC group

Differential analysis of significantly abundant bacteria among the groups revealed specific bacteria markers that characterize each group. Compared to the DSS group, resveratrol treatment was associated with the restoration of overpopulated genera such as *Provotella 9, Lachnoclostridium, Ruminococcaceae UCG 005*, and *Serratia* (Fig. 3A–E). Other overpopulated genera reduced by resveratrol included *Ruminoclostridium 5, Stenotrophomonas, Eubacterium fissicatena group*, and *Acinetobacter* (Fig. 3A). Further analysis revealed increased potential pathogenic genera in the DSS group, including *Lachnoclostridium, Acinetobacter*, and *Serratia*, that significantly differentiated the DSS group from the NC group (Fig. 3F) but failed to differentiate the RSV group from the NC groups (Fig. 3H). Earlier studies have identified *Lachnoclostridium* to be significantly enriched in the fecal samples of colitis [19], colitis-associated colorectal cancer [20] in mice, and as a potential marker in adenoma patients [21]. Moreover, *Acinetobacter* is enriched in actively inflamed colitis tissue [22] and Serratia has been long reported as an opportunistic pathogen [23]. Our results also showed that the RSV group had an increased abundance of ‘beneficial’ genera such as *Rikenellaceae RC9 gut group, Ruminococcaceae NK4A214 group, Ruminococcaceae UCG-005, Ruminiclostridium 5,* and *[Eubacterium] fissicatena* group compared with the NC group (Fig. 3H). Thus, resveratrol treatment not only decreases the overpopulation of bacteria groups but also increases certain beneficial species. The implication of these modulations on differential gut microbiota functional changes was examined via the KEGG heatmap functional prediction analysis and KEGG LFfSe LDA (Fig. 3G, I). The comparison between the DSS and NC groups revealed eight significantly dysregulated functions in the DSS group, including downregulated amino acid metabolism, metabolism of terpenoids and polyketides, and signaling molecules and interaction, but upregulated transcription and carbohydrate metabolism (Fig. 3I). Interestingly, KEGG LFfSe LDA revealed no significantly differential dysregulated function between the RSV group and the NC group, implicating the ability of resveratrol to restore colitis-induced functional alterations of the microbiota.

### Resveratrol reduces dysregulation of gut metabolites in the mitigation of colitis

**Fig. 4:**
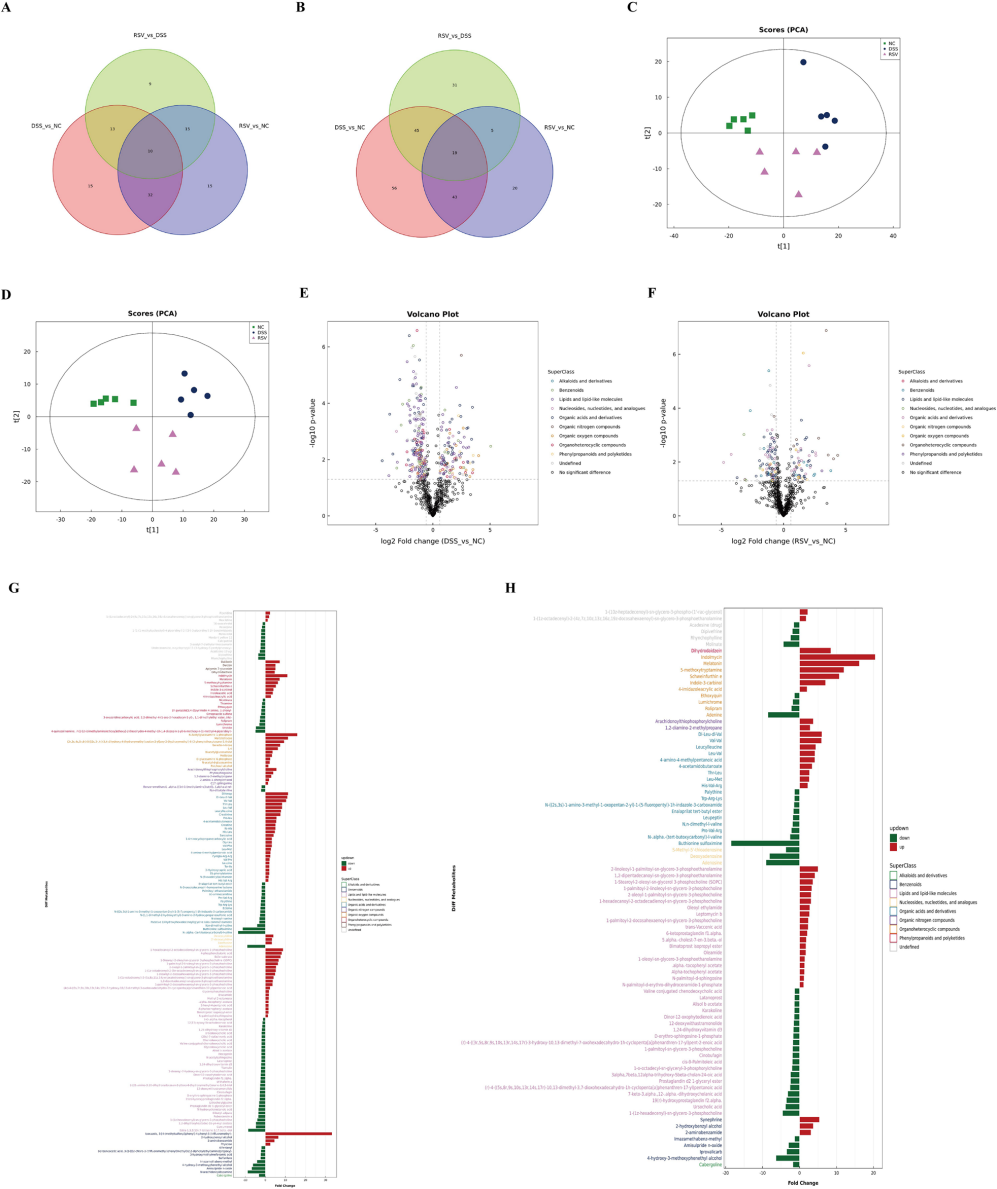
Resveratrol reduces gut metabolites dysregulation. **A** Venn diagram of differential metabolites within different groups in negative ion mode. **B** Venn diagram of differential metabolites within different groups in positive ion mode. **C** PCA score map in negative ion mode. **D** PCA score map in positive ion mode. **E** Volcano plot of differential metabolites between the DSS and NC groups in positive ion mode. **F** Volcano plot of differential metabolites between the RSV and NC groups in positive ion mode. **G** Multiple analysis of significant differences in metabolite expression in positive ion mode between the DSS and NC groups. **H** Multiple analysis of significant differences in metabolite expression in positive ion mode between the RSV and NC groups

Gut microbiota-derived metabolites play a crucial role in the onset and development of many diseases, including IBD [24–26]. Our earlier studies revealed that gut metabolites are significantly altered between healthy controls and IBD in both humans and mice [27, 28]. Using the UHPLC/Q-TOF–MS technique, samples were quality controlled (Additional file 1: Fig. S1B–E) and a total of 1163 metabolites belonging to 12 superclass chemical classifications (Additional file 1: Fig. S1A) were identified in the current study. While 233 of these metabolites were dysregulated between the DSS vs NC groups, this was reduced to 147 between the RSV vs NC groups (Fig. 4A, B). Relative to the control group, analysis of differential metabolites in the positive ion mode revealed a reduced number of dysregulated metabolites in the resveratrol-treated group (87 metabolites) than the untreated group (163 metabolites) (Fig. 4B). PCA was used to help observe the cluster location of metabolites in each group. Results showed a reduced PCA score in the DSS group compared to the NC group, with the RSV group’s score located between the NC and DSS groups in both positive and negative ion modes (Fig. 4C, D). Volcanic plot analysis revealed a decrease in the concentration of significantly differential metabolites between the RSV vs NC groups as compared to the DSS vs NC groups (Fig. 4E, F). An intuitive expression of all differential metabolites classified into their respective superclass in the positive ion mode between the DSS vs NC and RSV vs NC groups are presented in Fig. 4G, H.

### Resveratrol modulates the arginine and proline metabolic pathway in the mitigation of colitis

**Fig. 5:**
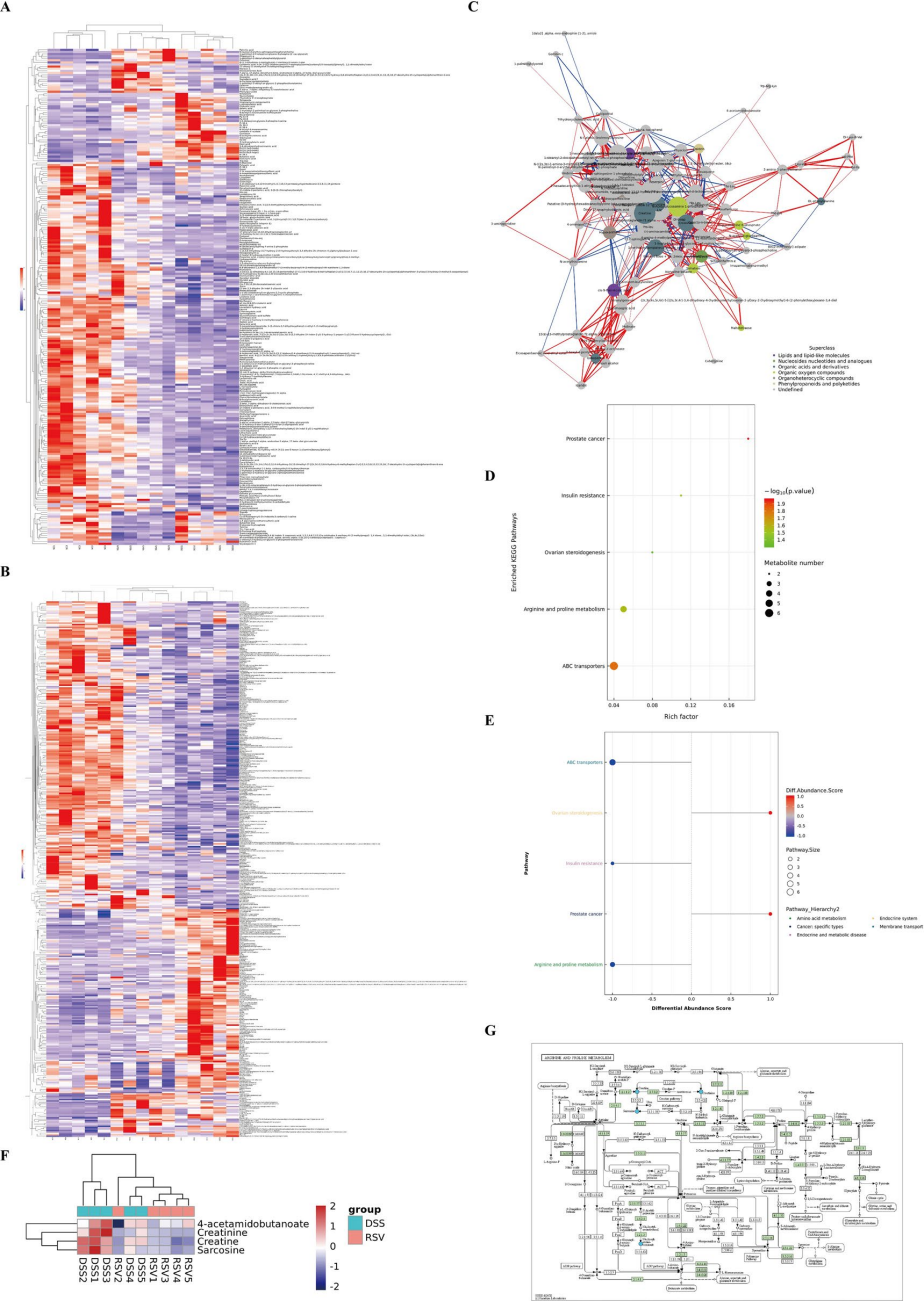
Resveratrol modulates the arginine/proline metabolic pathway. **A** Hierarchical clustering heatmap of significantly differential metabolites in negative ion mode. **B** Hierarchical clustering heatmap of significantly differential metabolites in positive ion mode. **C** Differential metabolite network diagram between the RSV group and the DSS group in positive ion mode. **D** KEGG enrichment pathway diagram (bubble diagram). **E** Differential abundance score plot of all enriched metabolic pathways between the RSV group and the DSS group. **F** Clustering heatmap of differential metabolites in the KEGG pathway for arginine and proline metabolism between the DSS and the RSV groups. **G** KEGG pathway diagram of arginine and proline metabolism containing differential metabolites

We further performed functional analysis of the differentially expressed metabolites through hierarchical clustering heatmap, network, and KEGG pathway analyses to determine key metabolites and their associated pathways crucial in the resveratrol-mediated mitigation of colitis. The clustering heatmap showed unique clusters of differential metabolites between the three groups (Fig. 5A, B, Additional file 1: Fig. S1I, J) while the correlation network showed the interaction between six defined superclasses of molecules between the resveratrol treated and untreated groups, including lipids and lipid-like molecules, organic acids and derivatives, organoheterocyclic compounds, and organic oxygen compounds (Fig. 5C). Among the key molecules at the center of the network was creatine and its derivative, creatinine, which belongs to organic acids and derivatives. KEGG enrichment pathway analysis between the resveratrol-treated (RSV) and untreated (DSS) groups revealed the two most enriched metabolic pathways as arginine and proline metabolism and ABC transporters (Fig. 5D), which was further confirmed with differential abundance score plot (Fig. 5E). Concerning the arginine and proline metabolic pathway, creatine, creatinine, and two other molecules (sarcosine and 4-acetamidobutanoate) were the differentially significant molecules expressed between the RSV and DSS groups (Fig. 5F), where resveratrol treatment reduced their overexpression. The detailed connection between these molecules in arginine and proline metabolism and other related metabolic pathways is shown in Fig. 5G. These findings implicate the microbiota-arginine/proline metabolism axis in the treatment effect of resveratrol in colitis.

### Resveratrol modulates the microbiota-arginine/proline metabolic axis during the mitigation of colitis

**Fig. 6:**
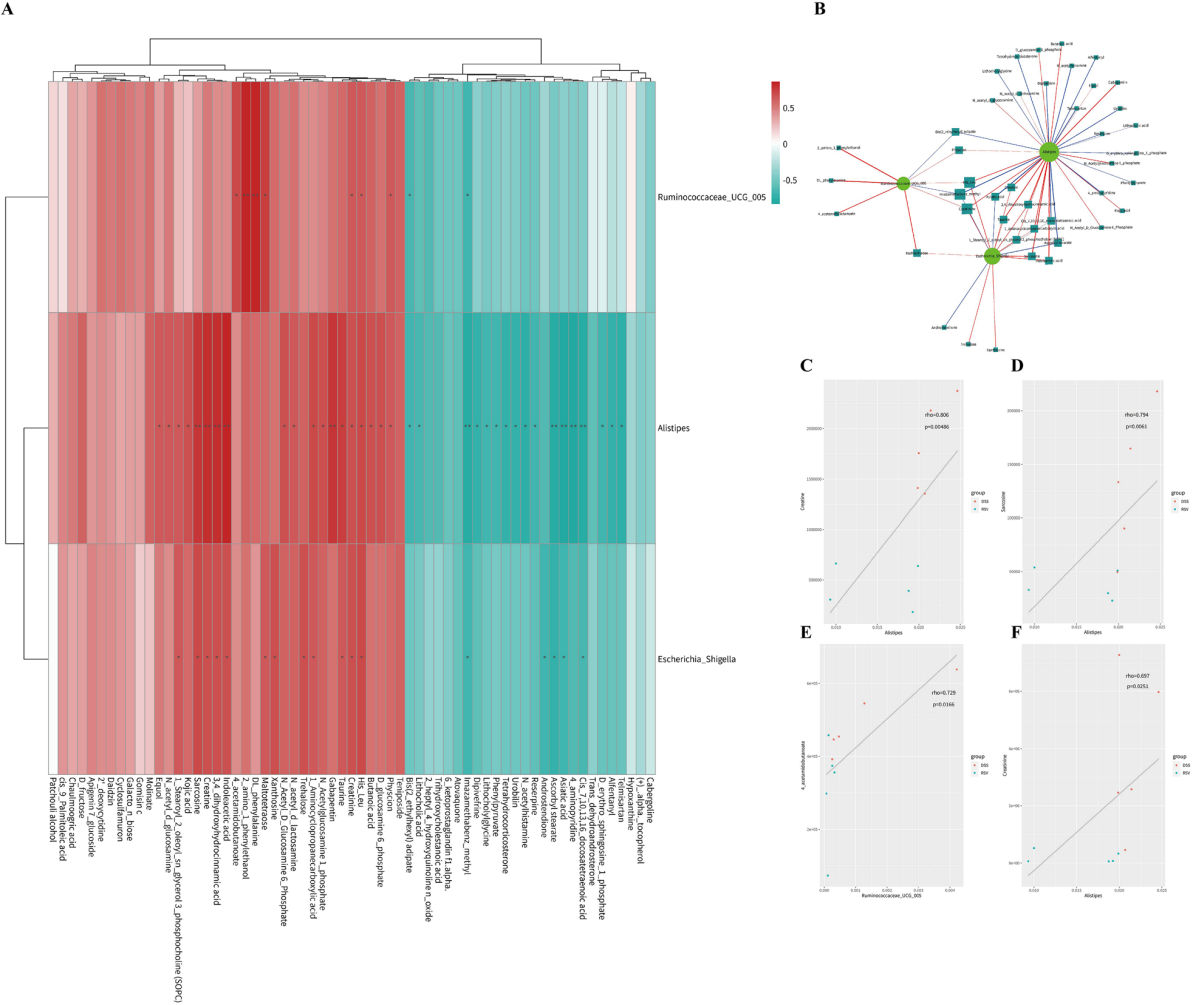
Resveratrol modulates the microbiota-arginine/proline metabolic axis. **A** Spearman correlation analysis of hierarchical clustering heatmap of significant difference microbiota and significant difference metabolites betweenthe RSV and DSS groups. *P*-value reflects the significant level of correlation and was defined by *P* < 0.05 as *, *P* < 0.01 as **, *P* < 0.001 as ***. **B** Spearman correlation analysis network of significant difference flora and metabolites between the RSV and DSS groups. In the correlation network diagram, the color of the line represents the positive and negative value of the correlation coefficient between the two (blue represents negative correlation and red represents positive correlation), and the thickness of the line is directly proportional to the absolute value of the correlation coefficient. The node size is positively correlated with its degree, that is, the greater the degree, the larger the node size; **C**–**F** Representative scatter diagram of correlation (Creatine, Sarcosine,4-acetamidobutanoate, Creatinine)

Having established the ability of resveratrol to modulate the microbiota and its metabolites during the mitigation of colitis, correlation analysis of the significantly differential microbiota and metabolites was performed to assess their interaction. Between the RSV and DSS groups, three bacteria genera (*Ruminococcaceae UCG 005, Alistipes,* and *Escherichia/Shigella*) and 63 metabolites were correlated (Fig. 6A), and a network diagram between the three bacteria genera and 42 metabolites was constructed (Fig. 6B). It was revealed that creatinine significantly correlated with all three bacteria genera, creatine and sarcosine correlated with two genera, and 4-acetamidobutanoate correlated with one genus. This indicates potential interaction points between the arginine and proline metabolic pathway and these gut bacteria genera. A scatter plot further showed the significant regulatory effect of resveratrol on the arginine and proline metabolism-associated molecules (Fig. 6C–F), which could also serve as potential markers for successful resveratrol-treated colitis.

### Resveratrol regulates arginine metabolism in mice colon mucosal tissue in the mitigation of colitis

**Fig. 7:**
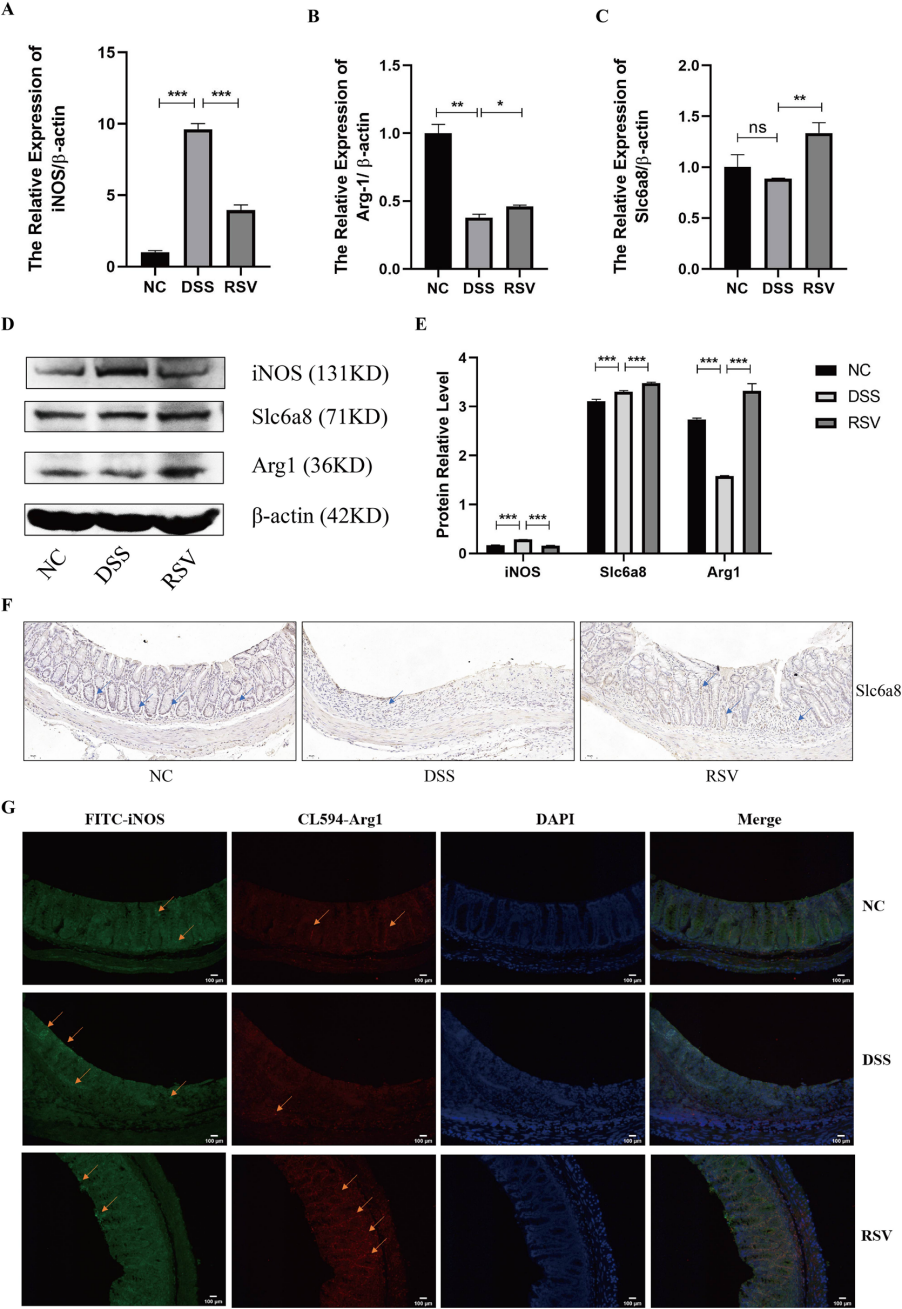
Resveratrol regulates arginine metabolism in mice colon. **A**–**C** qRT-PCR analysis of the mRNA expression level of the arginine metabolism-related molecules (iNOS, Arg1, Slc6a8) in mouse colon tissues. **D**–**E** Western blot analysis of the protein expression level of arginine metabolism-related molecules in mouse colon tissues and its grayscale scanning analysis. **F** IHC analysis of Slc6a8 expression in mouse colon tissues (200 ×). **G** Representative images of IF staining for iNOS and Arg1 on sections of mouse colon tissues (200 ×). * represents *p* < 0.05, ** represents *p* < 0.01 and *** represents *p* < 0.001

After establishing the potential role of the arginine and proline metabolic axis in the regulatory function of resveratrol during the mitigation of colitis through the colonic cytokine expression, tissue examination, as well as metagenomics and metabolomics analysis of the mice fecal samples, the specific mechanism involved in this effect was explored using mice colonic mucosal tissues. Previous studies indicate that macrophages can metabolize arginine in two major ways, i.e., breakdown arginine into iNOS to promote inflammation or produce polyamines via Arg1 to promote anti-inflammation [11, 29]. In a related process, Slc6a8, a creatine transporter highly expressed by macrophages, participates in anti-inflammatory effects by transporting creatine [11]. Therefore, we examine whether resveratrol could upregulate Slc6a8 to transport creatine into macrophages and enhance the metabolism of the Arg1 pathway, thereby reducing the iNOS-induced inflammatory response in the gut. qRT-PCR analysis of mice colon tissues showed reduced expression of iNOS but increased Slc6a8 and Arg1 in the resveratrol-treated mice (RSV group) compared to the untreated (DSS) group (Fig. 7A–C). The same expression pattern was confirmed by western blot analysis (Fig. 7D, E) while immunohistochemistry revealed significantly restored levels of Slc6a8 in RSV tissues compared to DSS tissues (Fig. 7F). Moreover, the immunofluorescence technique was used to confirmed the reduced expression of iNOS and increased levels of Arg1 in the resveratrol treated group compared to the DSS group (Fig. 7G). These observations implicate that resveratrol relieves colitis by not only inhibiting iNOS but also promoting the crucial enzyme Arg1 and the transporter Slc6a8.

### *Resveratrol regulates arginine metabolism in macrophages *in vitro

**Fig. 8:**
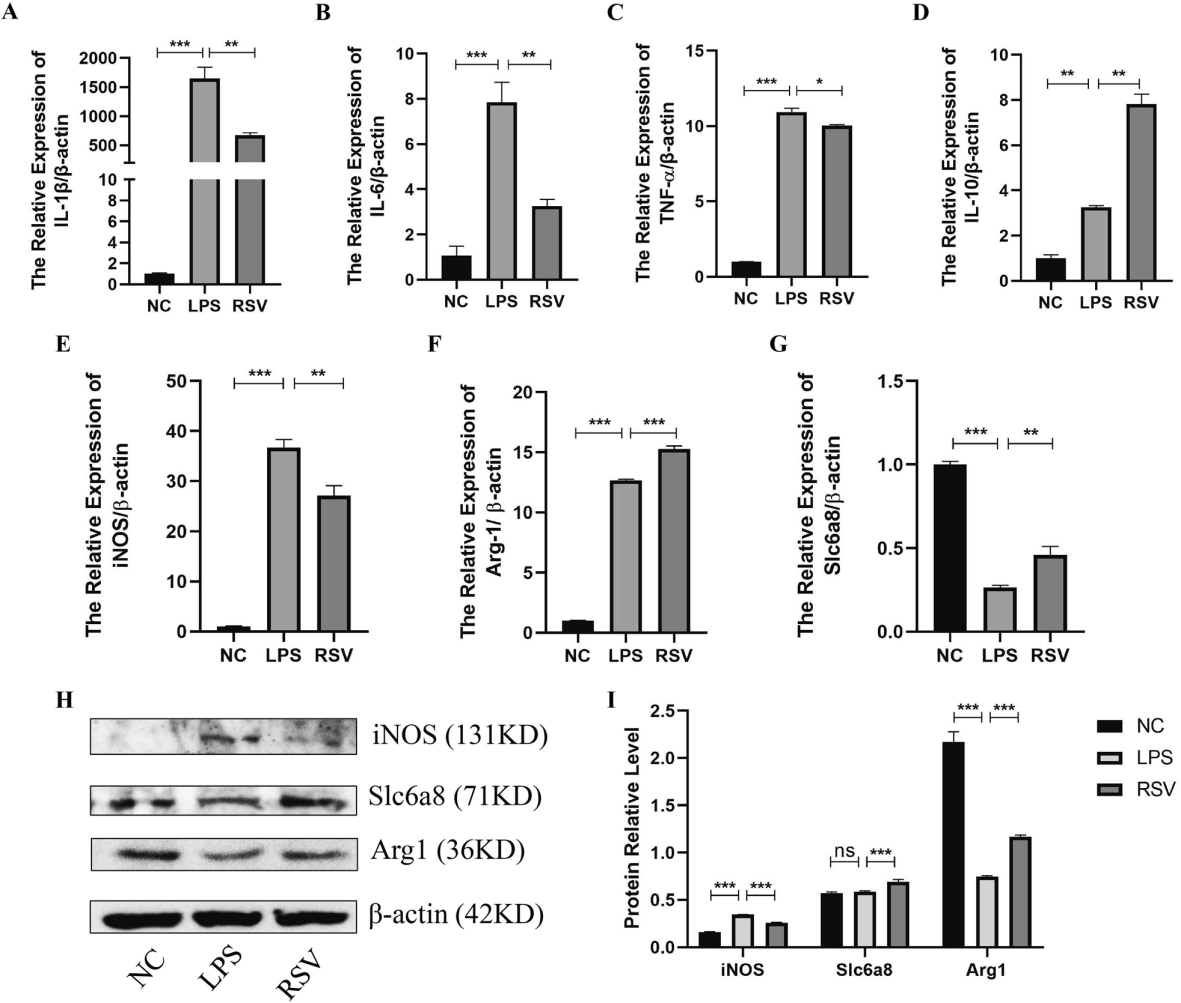
Resveratrol regulates arginine metabolism in macrophages in vitro. **A**–**D **qRT-PCR detection of cytokines expression in RAW264.7 cells. **E–G** qRT-PCR analysis of the mRNA expression level of the mRNA level of arginine metabolism-related molecules (iNOS, Arg1, Slc6a8) in RAW264.7 cells. **H**, **I** Western blot analysis of the protein expression level of arginine metabolism-related molecules in RAW264.7 cells and its grayscale scanning analysis. * represents *p* < 0.05, ** represents *p* < 0.01 and *** represents *p* < 0.001

Restoring colonic immune balance by targeting macrophage polarization is crucial to mitigating colitis. The anti-inflammatory effect of macrophages is linked with arginine metabolism through increased macrophage Arg1 activity and reduced iNOS expression. After establishing the ability of resveratrol to increase Arg1 and Slc6a8 and decrease iNOS in the colonic mucosa of mice, we examined whether this effect was associated with macrophages. Therefore, we cultured RAW264.7 macrophage cell line in an LPS-induced inflammatory environment and treated it with 80 nmol/ml resveratrol for 12 h. Results of qRT-PCR showed the anti-inflammatory effect of resveratrol on macrophages as it downregulated the pro-inflammatory cytokines IL-1β, IL-6, and TNF-α but upregulated the anti-inflammatory cytokine IL-10 (Fig. 8A–D). As observed in the colonic mucosa of the mice model, resveratrol significantly downregulated iNOS and upregulated Arg1 and Slc6a8 in macrophages compared to the untreated setup (LPS) (Fig. 8E–G). Western blot confirmed the same trend of increased Arg1 and Slc6a8 and decreased iNOS in resveratrol-treated macrophages (Fig. 8H, I). These findings indicate the ability of resveratrol to relieve inflammation through the modulation of the macrophage-arginine metabolism axis.

## Discussion

IBD is characterized by chronic inflammation associated with immune dysregulation and dysbiosis. Resveratrol, a natural plant compound with anti-inflammatory and antioxidant properties, has been shown to possess therapeutic efficacy in several conditions, including IBD [30], metabolic syndromes [31], aging and age-related diseases [32], wound healing [33], vascular system conditions [34], and potentially, as a cancer therapy [35]. In this study, our initial assessment revealed the ability of resveratrol to abrogate colitis by reducing colonic pro-inflammatory markers (IL-1β, IL-6, TNFα) and DAI of mice, increasing anti-inflammatory marker IL-10, increasing colon tight junction molecules (Occludin, Claudin 1), and enhancing colon and spleen tissue repair and gross appearance. Other studies report that resveratrol reduces inflammation in patients with UC to improve their quality of life and disease clinical colitis activity [36], and in animal models of IBD [14, 37], where there are reduced pro-inflammatory markers (TNF-α, IFN-γ, IL-1β, IL-6, and IL-4) and DAI. In their randomized, double-blind, placebo-controlled pilot study, Maryam and colleagues found that the supplementation of 500 mg resveratrol daily for 6 weeks in active mild to moderate UC patients significantly reduced inflammatory markers such as plasma levels of TNF-α, high-sensitivity C-reactive protein (hs-CRP), and activity of NF-κB in PBMCs (peripheral blood mononuclear cells) [36]. The DSS-induced enteritis model in mice is very similar to human UC in terms of clinical symptoms and pathological features. However, due to species and environmental factors, we cannot fully simulate the human pathogenesis, which is also one of the shortcomings of this study, but it can still provide new ideas for the exploration of pathogenesis.

The gut microbiota is a central regulator of not only the host immune system, metabolism, and development but also influences the onset and development of several diseases, including IBD. Recent developments in genome sequencing technologies, bioinformatics, and culturomics have enabled researchers to explore the microbiota at a more detailed level than before, and in particular, their functions and association with disease and health [38]. We used 16S rDNA gene sequencing of the microbiome in fecal samples to assess the microbial changes triggered by resveratrol in the mitigation of colitis. Compared to the colitis untreated group, resveratrol treatment improved colitis-induced microbiota changes in the OTU structure and diversity. Specifically, resveratrol not only restored the alpha and beta diversity but reversed inflammation-induced changes in the abundance of the three most dominant phyla (*Bacteroidetes, Proteobacteria, Firmicutes*), thus significantly restoring the Bacteroidetes/Firmicutes ratio, a crucial factor in maintaining normal intestinal homeostasis [39, 40]. Resveratrol reduced the overpopulated of several bacteria genera, including potential pathogenic genera such as *Lachnoclostridium, Acinetobacter*, and *Serratia*, and increased ‘beneficial’ genera such as the *Rikenellaceae RC9 gut group, Ruminococcaceae NK4A214 group, Ruminococcaceae UCG-005, Ruminiclostridium 5*, and *[Eubacterium] fissicatena* group. Moreover, while eight microbiota-associated functions were significantly dysregulated in the untreated colitis mice, including amino acid metabolism, carbohydrate metabolism, transcription, and signaling molecules and interaction, KEGG LFfSe LDA revealed no significantly dysregulated function in the resveratrol-treated mice compared to the normal controls. This indicates that resveratrol potently mitigates diseases by improving microbiota and functional alterations in the gut as documented in experimental diseases like colitis [41, 42], obesity [43], diabetic nephropathy [44], and atherosclerosis [45], among others. Concerning colitis, studies have shown that more RSV tends to be metabolized in the intestinal flora after oral administration, and that mice with different gut microbiota composition metabolize RSV differently. Our study indicated that the intake of RSV alters the intestinal microbiota of DSS-induced mice. Moreover, the amount of metabolites such as dihydro-resveratrol increased accordingly, suggesting a specific role of RSV in the regulation of intestinal flora and its metabolites. In another study, the probiotic strain Li01 promoted larger amounts of RSV metabolizing into DHR, RES-sulfate, and RES-glucuronide, where the level of DHR increased most significantly among the metabolites [46]. Due to the extent of the exploration in this study, we only focused on RSV as a potential treatment for colitis, however, other compounds with similar properties could elicit similar responses. Thus, presenting a gap to be researched in future studies.

One of the primary means through which the gut microbiota interacts with the host is employing metabolites, which are small molecular particles produced as intermediate or end products of microbial metabolism [24]. Signals from microbial metabolites influence immune homeostasis, immune maturation, host energy metabolism, and maintenance of mucosal integrity, thus, alterations in the composition and function of the microbiota and its derived metabolites are associated with several diseases, including IBD [24–26]. In this study, fecal metabolite detection via UHPLC/Q-TOF–MS indicated that resveratrol treatment reduced the number of significantly dysregulated metabolites from 233 (DSS vs NC) to 147 (RSV vs NC). The KEGG enrichment pathway analysis revealed that the arginine–proline metabolism axis was crucial in the resveratrol-mediated mitigation of colitis, as resveratrol treatment reduced the overexpression of molecules in this axis (creatine, creatinine, sarcosine, and 4-acetamidobutanoate). Arginine metabolism is crucial in the pathophysiology of IBD, as its components, the arginine–creatine and arginine–polyamine axis are mainly protective against inflammation, relative to the pro-inflammatory arginine–nitric oxide (iNOS) axis [47]. In colon mucosa samples, we found that resveratrol not only increase Arg1 and the creatine transporter Slc6a8, which together promote anti-inflammation but also decreased iNOS. Slc6a8, which is confirmed to significantly decrease in both CD and UC, is known to regulate energy balance in intestinal epithelial cells and thereby epithelial integrity and barrier function [48]. In IBD patients, increased sarcosine is reported as a metabolomic marker linked with relapse of quiescent CD and UC [49], while 4-acetamidobutanoate is associated with subjects with high liver and kidney disease severity [50]. Therefore, the potent modulation of arginine metabolism is promising in IBD treatment and requires further exploration.

Macrophages are key in the resolution of gut inflammation and are linked with arginine metabolism through the expression of the arginine catalytic enzymes Arg1 and iNOS, and the generation of creatine as a key source of cellular energy reserve [11]. Our results showed that resveratrol downregulated macrophage expression of iNOS alongside the pro-inflammatory cytokines IL-1β, IL-6, and TNF-α but upregulated Arg1, Slc6a8, and the anti-inflammatory cytokine IL-10. Creatine reprograms macrophage polarization by inhibiting immune effector molecules such as iNOS and IFN-γ-JAK-STAT1 transcription-factor signaling while enhancing IL-4-STAT6-activated Arg1 expression. Depletion of intracellular creatine by reduced expression of Slc6a8 alters macrophage-mediated immune responses [11]. The ability of resveratrol to regulate all three key metabolic fates of arginine towards anti-inflammation might have significantly contributed to relieving inflammation in vivo and in vitro. However, the interaction between RSV and gut microbiota involves a very complex mechanism. Our study found that arginine metabolism plays an important role in this process, which only provides a new perspective and idea, and the detailed mechanism still needs to be further explored.

**Fig. 9:**
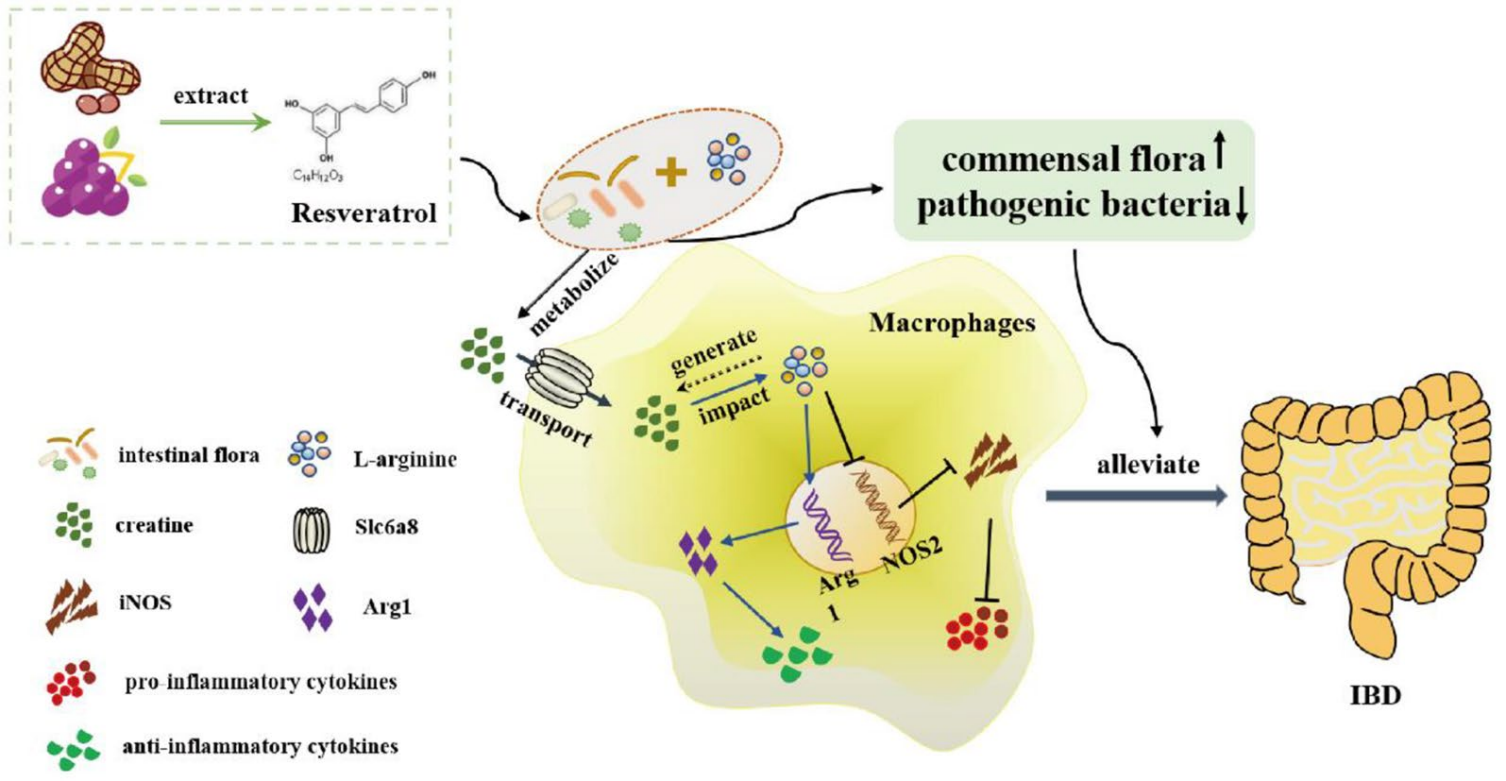
Summary of the mechanism of resveratrol-mediated colitis

Mechanistically, resveratrol increases the expression of Slc6a8 on macrophages to transport sufficient amounts of creatine. The intracellular creatine influences arginine metabolism towards upregulated production of the Arg1 gene and decreased production of the NOS2 gene. Thus, there is decreased secretion of pro-inflammatory factors and increased anti-inflammatory factors, resulting in improved microbial community composition, diversity, and metabolites, and ultimately relieving colitis (Fig. 9).

## Conclusions

Resveratrol mitigates colitis by relieving clinical features of colitis, increasing tight junction molecules, improving microbiota composition and diversity, and reducing metabolite dysregulation in mice. This effect is associated with arginine metabolism-mediated macrophage polarization through the upregulation of Arg1 and Slc6a8, and the downregulation of iNOS. This uncovers a previously unknown mechanism of resveratrol in the treatment of IBD and provides the microbiota-macrophage-arginine metabolism axis as a potential therapeutic target for gut inflammation.

### Supplementary Information

**Additional file 1: Figure S1. A** The number of identified metabolites in each chemical classification. **B** PCA analysis of negative ion mode population samples and quality control samples. **C** PCA analysis of positive ion mode population samples and quality control samples. **D** Correlation map of QC samples in negative ion mode. **E** Correlation map of QC samples in positive ion mode. **F** Rank abundance curve reflecting species abundance and uniform distribution of species. **G** Shannon curve indicating that the amount of sequencing data is large enough to reflect the vast majority of microbial information in the samples. **H** Species accumulation curve on the adequacy of sample size and estimation of species richness. **I** KEGG Hierarchical Clustering Analysis of expression changes within the groups in negative ion mode. **J** KEGG Hierarchical Clustering Analysis of expression changes within the groups in positive ion mode.

## Data Availability

The datasets used and/or analyzed during the current study are available from the corresponding author upon reasonable request.

## Abbreviations

IBD: Inflammatory bowel disease
UC: Ulcerative colitis
CD: Crohn’s disease
IFN-γ: Interferon γ
TNF-α: Tumor necrosis factor-alpha
LPS: Lipopolysaccharide
IL: Interleukin
Arg1: Arginase 1
iNOS: Inducible nitric oxide synthase
Slc6a8: Solute carrier family 6 member 8
UHPLC/Q-TOF–MS: Ultra-high-performance liquid chromatography-quadrupole time-of-flight mass spectrometry
IHC: Immunohistochemistry
DSS: Dextran sulfate sodium
DAI: Disease activity index
PCNA: Proliferating cell nuclear antigen
hs-CRP: High-sensitivity C-reactive protein
PBMCs: Peripheral blood mononuclear cells
