# Supplementary material for: Resveratrol alleviates DSS-induced IBD in mice by regulating the intestinal microbiota-macrophage-arginine metabolism axis
Source: Eur J Med Res. 2023 Sep 2;28:319. doi: 10.1186/s40001-023-01257-6 (PMC10474707; doi:10.1186/s40001-023-01257-6)
Supplement: Supplementary file 1 — Additional file 1: Figure S1. A The number of identified metabolites in each chemical classification. B PCA analysis of negative ion mode population samples and quality control samples. C PCA analysis of positive ion mode population samples and quality control samples. D Correlation map of QC samples in negative ion mode. E Correlation map of QC samples in positive ion mode. F Rank abundance curve reflecting species abundance and uniform distribution of species. G Shannon curve indicating that the amount of sequencing data is large enough to reflect the vast majority of microbial information in the samples. H Species accumulation curve on the adequacy of sample size and estimation of species richness. I KEGG Hierarchical Clustering Analysis of expression changes within the groups in negative ion mode. J KEGG Hierarchical Clustering Analysis of expression changes within the groups in positive ion mode. [file 40001_2023_1257_MOESM1_ESM.docx]

**Additional file 1**


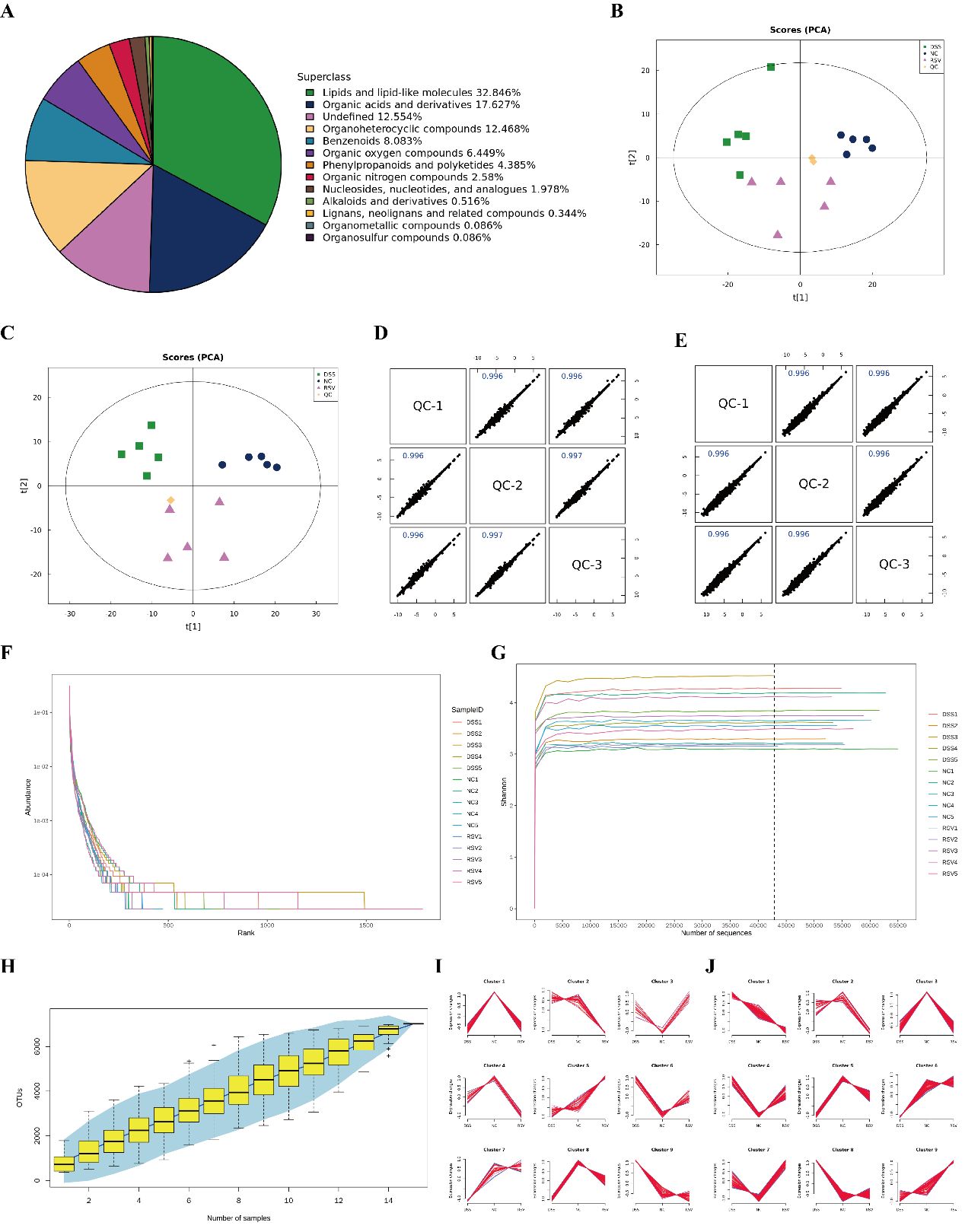


**Figure S1.** (A) The number of identified metabolites in each chemical classification. (B) PCA analysis of negative ion mode population samples and quality control samples. (C) PCA analysis of positive ion mode population samples and quality control samples. (D) Correlation map of QC samples in negative ion mode. (E) Correlation map of QC samples in positive ion mode. (F)Rank abundance curve reflecting species abundance and uniform distribution of species. (G) Shannon curve indicating that the amount of sequencing data is large enough to reflect the vast majority of microbial information in the samples. (H) Species accumulation curve on the adequacy of sample size and estimation of species richness. (I) KEGG Hierarchical Clustering Analysis of expression changes within the groups in negative ion mode. (J) KEGG Hierarchical Clustering Analysis of expression changes within the groups in positive ion mode.
